# Montelukast attenuates interleukin IL-1β-induced oxidative stress and apoptosis in chondrocytes by inhibiting CYSLTR1 (Cysteinyl Leukotriene Receptor 1) and activating KLF2 (Kruppel Like Factor 2)

**DOI:** 10.1080/21655979.2021.1984003

**Published:** 2021-10-25

**Authors:** Zongwei Li, Jianming Wang, Yumin Ma

**Affiliations:** a School of Pharmaceutical Engineering, Guangdong Food and Drug Vocational College, Guangzhou City, Guangdong Province, China; b Department of Pharmaceutical Machinery, Maternal and Child Health and Family Planning Technical Service Center, Wuwei City, Gansu Province, China

**Keywords:** Montelukast, CysLTR1, KLF2, osteoarthritis

## Abstract

Montelukast is a cysteinyl leukotriene receptor 1 (CysLTR1) antagonist widely used to suppress the inflammatory response in asthma and allergic rhinitis. This study aimed to investigate the potential impacts of montelukast on osteoarthritis (OA) progression. To determine the role of montelukast in OA, the expression of CysLTR1 was first examined by quantitative reverse transcription PCR (RT-qPCR) and western blot in IL-1β-induced ATDC5 cells treated with or without montelukast. Subsequently, the impacts of montelukast on cell viability and oxidative stress were measured by Cell-Counting-Kit-8 (CCK-8), commercial kits and western blot. Oxidative stress-related protein expressions were determined by western blot analysis in Il-1β-induced ATDC5 cells. Cell apoptosis and cartilage degradation were examined by TdT-mediated dUTP Nick-End Labeling (TUNEL) assay, western blot and RT-qPCR. KLF2 expression was measured in IL-1β-induced ATDC5 cells treated with montelukast. After interference with small interfering RNA (siRNA)-KLF2 in ATDC5 cells, the loss-of-function assays were also performed in same ways. CysLTR1 expression was elevated in IL-1β-induced ATDC5 cells but inhibited significantly by montelukast. Montelukast attenuated the oxidative stress and apoptosis, improved cell viability. Moreover, montelukast enhanced KLF2 expression. After transfected with siRNA-KLF2, montelukast attenuated cell injury, oxidative stress, apoptosis and cartilage degradation in IL-1β-induced ATDC5 cells by activating KLF2.In summary, this work elaborates the evidence that montelukast could attenuate oxidative stress and apoptosis in IL-1β-induced chondrocytes by inhibiting CysLTR1 and activating KLF2, which can guide the therapeutic strategies of montelukast for OA development in the future.

## Introduction

OA is a chronic, debilitating joint disease that influences millions of people in the world [1]. With few reliable treatments available for OA, it has become an arguably complicated disease whose etiology crosses biomechanics and biochemistry and whose prevalence rapidly increases with age and sex-specific differences [1–3]. OA is probably the final result of joint damage stimulated by a wide range of biomechanical and metabolic factors, including such as interleukin- (IL-)1β, IL-6, and tumor necrosis factor- (TNF-) α [4]. Difficulty in making sense of the phenotype of OA is inextricably linked to the huge obstacles in identifying OA-related genes [5]. Thus, experts are switching their attention to finding novel drugs that are available for effective OA treatment. IL-1β plays a central role in the pathogenesis of OA, which is involved in chondrogenic extracellular matrix synthesis and the expression of matrix metalloproteinases, as well as the expression of inflammatory mediators and apoptosis [6–10]. In this study, IL-1β was used to stimulate chondrocytes to establish cell model of OA.

Montelukast is generally deemed as a safe drug with only a few adverse drug reactions [11,12]. It is a potent selective antagonist of leukotriene D4 at the cysteinyl leukotriene (cysLT) type 1 receptor, which is presented in human respiratory epithelial cells and is produced by cells including macrophages and eosinophils [12,13]. Montelukast is also well documented as a critical anti-inflammatory modulator widely used for asthma and allergic rhinitis treatment, and its anti-oxidant properties in multiple tissues and organs cannot be overlooked [14,15]. Furthermore, montelukast treatment protects the liver against LPS-induced oxidative damage [16]. Chronic nonproductive cough can be cured with montelukast. Additionally, it has been shown to retard the cell senescence of chondrocytes induced by tumor necrosis factor-α [17]. To our interest, montelukast suppresses receptor activator of nuclear factor‑κB ligand-induced osteoclast formation and bone loss via CysLTR1 and P2Y12 [18]. Nevertheless, an improved understanding of the role of montelukast in inflammation and apoptosis of chondrocytes remains to be achieved.

Chondrocytes are the mere cells located in articular cartilage and are involved in the initiation of OA [19–22]. Previous report has indicated a tight relationship between chondrocyte apoptosis and articular erosion [23]. Taken together, we predict that montelukast could play a protective role in ameliorating oxidative stress and apoptosis in OA. Therefore, in this paper, we established an IL-1β- induced *in vitro* OA model to observe the potential role of montelukast in oxidative stress and apoptosis in IL-1β-induced chondrocytes and reveal its underlying mechanism in OA.

## Materials and methods

### Cell line and treatment

The chondrocytes (ATDC5) purchased from Cell Bank of Chinese Academy of Sciences (Shanghai, China) were cultured in DMEM supplemented with 10% fetal bovine serum, penicillin (100 U/ml) and streptomycin (100 g/ml) at 37°C in a humidified incubator with 5% CO_2_.

To establish an *in vitro* OA model, cells were treated with IL-1β (10 ng/ml) [24–26] for 24 h. Then, 10 μm CysLTR1 antagonist (montelukast), which was bought from Cayman Chemical Company (Ann Arbor, MI, USA), was used to treat these cells for another 24 h. Cells were respectively transfected with KLF2 siRNA plasmid (siRNA-KLF2-1/2) or negative control plasmid (siRNA-NC) by the use of Lipofectamine 2000 reagent (Invitrogen) as per the operating protocols.

Cell viability assay

ATDC5 cells (4 × 10^5^ cells/well) were seeded into a 96-well plate. Cells were then treated with IL-1β and siRNA-KLF2-2 for 24, 48 and 72 h, following which 10 μL CCK-8 solution (Dojindo, Kumamoto, Japan) was added to each well for 4 h at 37°C. The absorbance of each well was measured by a microplate reader (Bio-Rad, Hercules, USA) at a wavelength of 450 nm.

### Quantitative Real-Time PCR

Total RNA was isolated from ATDC5 cells using TRIzol Reagent (Invitrogen, 120 Carlsbad, CA, USA) in keeping with the manufacturer’s protocols. Total RNA was then reverse transcribed to cDNA employing a RevertAid first-strand cDNA synthesis kit (Thermo Fisher Scientific, USA) conformed with the manufacturer’s recommendations. Quantitative real-time PCR was conducted with a SYBR Premix ExTaq kit (TaKaRa, Dalian, China) on an ABI 7500 Fast Real-Time PCR system (Applied Biosystems, USA). The mRNA levels were determined by threshold cycle (Cq) value and normalized against GAPDH expression. 2^−ΔΔCq^ method [27] was used for the quantification of qPCR results. The primer sequences used in this study are as following: CysLTR1 Forward: AAGTCCGTGGTCATAACCTTGT, Reverse: TCTGGGTACATAAGTCACGCT; KLF2 Forward: TTCGGTCTCTTCGACGACG, Reverse: TGCGAACTCTTGGTGTAGGTC; GAPDH: Forward: GGAGCGAGATCCCTCCAAAAT, Reverse: GGCTGTTGTCATACTTCTCATGG.

### Western blot

The treated ATDC5 cells were washed with PBS for three times, followed by the homogenization of total proteins in ice-cold lysis buffer. Then, cell debris was then centrifuged at 12,000 rpm at 4°C for 10 min. BCA kit was used for the quantification of protein levels. Equal amounts of extracted proteins were separated by 10% SDS-PAGE, and then transferred onto PVDF membranes. The membranes were covered with 5% skim milk for 30 min at room temperature, followed by an incubation with primary antibodies (anti-CysLTR1, PA5-28,735, ThermoFisher SCIENTIFIC) (anti- nuclear erythroid 2-related factor 2 (NRF2), ab137550; anti-Heme Oxygenase (HO-1), ab52947; anti-Bcl-2, ab32124; anti- apoptotic protease activating factor-1 (APAF-1), ab234436; anti-Bax, ab32503; anti-cleaved caspase3, ab32042; anti-cleaved poly ADP-ribose polymerase (PARP), ab32064; anti-KLF2, ab236507; anti-GAPDH, ab8245. England) overnight at 4°C. Subsequently, the membranes were incubated with the HRP-goat anti-rabbit secondary antibody (ab7090, abcam, England) for 1 h. The blots were visualized with the application of Bio-Rad ChemiDoc Imaging system (Bio-Rad, Hercules, CA, USA).

### ROS detection

The level of intracellular ROS was determined by a Reactive Oxygen Species Assay kit (Beyotime). Briefly, ATDC5 cells were harvested and stained with 10 μM DCF-DA in the dark at 37°C for 15 min. The cells were then washed by PBS for 3 times and immediately detected employing a flow cytometer (525 nm).

### Superoxide dismutase (SOD) and Malondialdehyde (MDA) detection

MDA level was measured by the thiobarbituric acid method, while SOD level was determined by the xanthine oxidase method. The procedures were performed in strict accordance with the recommendations provided by the manufacturer.

### TUNEL

Cell apoptosis was assessed using a TUNEL kit (KGA702-1, KeyGEN BioTECH, China) as per the manufacturer’s recommendations. ATDC5 cells were first washed with PBS, and then fixed in 4% paraformaldehyde for 4 h at 4°C. The cells were permeabilized in a medium containing 0.1% Triton X-100 for 2 min, followed by an incubation in TUNEL reaction mixture for 1 h at 37°C. Subsequently, the cells were washed with PBS for 3 times and counterstained with DAB staining solution for 15 min at room temperature. Images of apoptotic cells were captured under a fluorescence microscope.

### Statistical analysis

Data were expressed as mean ± standard deviation (SD), and analyzed using GraphPad Prism 7 (GraphPad Software Inc., La Jolla, CA, USA). Unpaired Student’s t-test was used for the comparisons of statistical significance between two groups and one-way ANOVA with turkey’s post hoc test for multiple comparisons. A p value < 0.05 was considered to show statistical significance.

## Results

Montelukast suppresses the expression of CysLTR1 in IL-1β-induced ATDC5 cells

**Figure 1.**
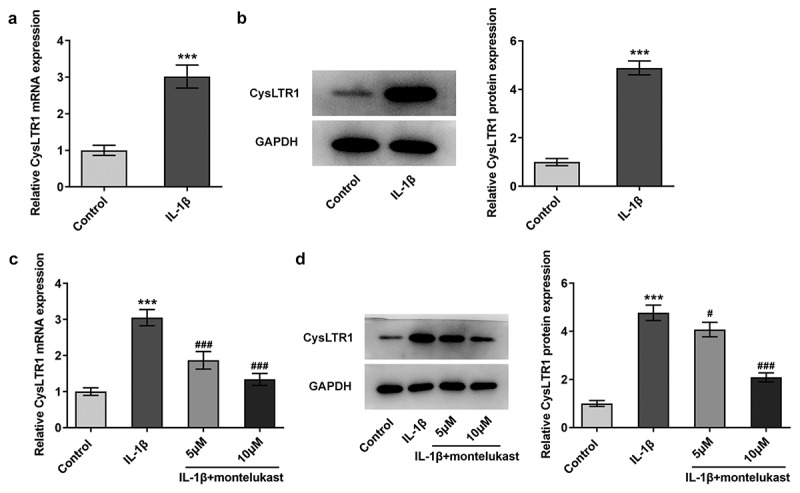
Montelukast suppresses the expression of CysLTR1 in IL-1β-induced ATDC5 cells. (a–b) The expression of CysLTR1 in ATDC5 cells induced by IL-1βwas detected by RT-qPCR and western blot. (c–d) The expression of CysLTR1 in IL-1β-induced ATDC5 cells was measured by RT-qPCR and western blot under montelukast treatment. ***p < 0.001 Vs control. ^#^p < 0.05, ^###^p < 0.001 Vs IL-1β

For the determination of the efficacy of montelukast on the development of OA, we first stimulated ATDC5 cells with the inflammatory factor IL-1β for 24 h to establish an OA *in vitro* environment. CysLTR1 in IL-1β-induced chondrocytes exhibited a remarkably elevated level as compared to that in control group (Figure 1(a,b)). Afterward, montelukast at the concentrations of 5 and 10 μm decreased the level of CysLTR1 in IL-1β-induced chondrocytes (Figure 1(c–d)). These results suggested that montelukast could suppress CysLTR1 expression in IL-1β-induced ATDC5 cells.

Montelukast attenuates the oxidative stress and apoptosis in IL-1β-induced ATDC5 cells

**Figure 2.**
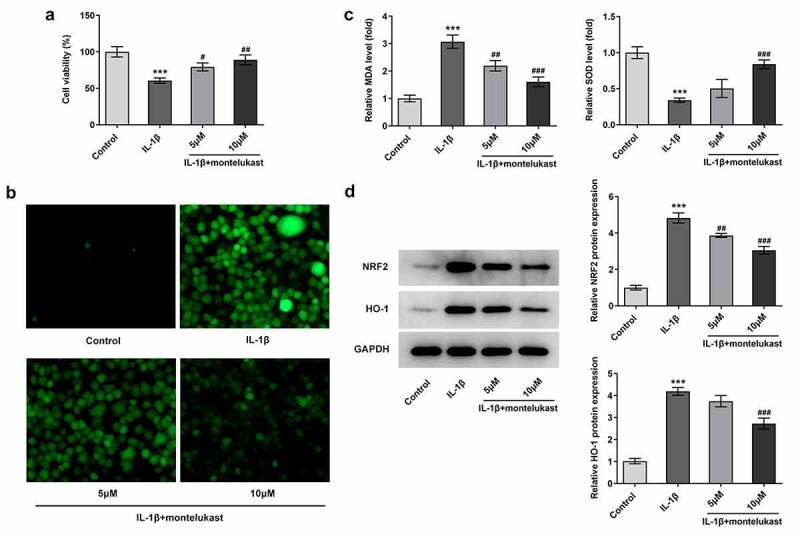
Montelukast attenuates the cell viability and oxidative stress in IL-1β-induced ATDC5 cells. (a) The cell viability of IL-1β-induced ATDC5 cells treated with montelukast was assayed by CCK-8. The (b) ROS, (c) MDA and SOD levels in IL-1β-induced ATDC5 cells treated with montelukast were measured by corresponding kits. (d) The expression of antioxidant proteins was measured by western blot. ***p < 0.001 Vs control. ^#^p < 0.05, ^##^p < 0.01, ^###^p < 0.001 Vs IL-1β

**Figure 3.**
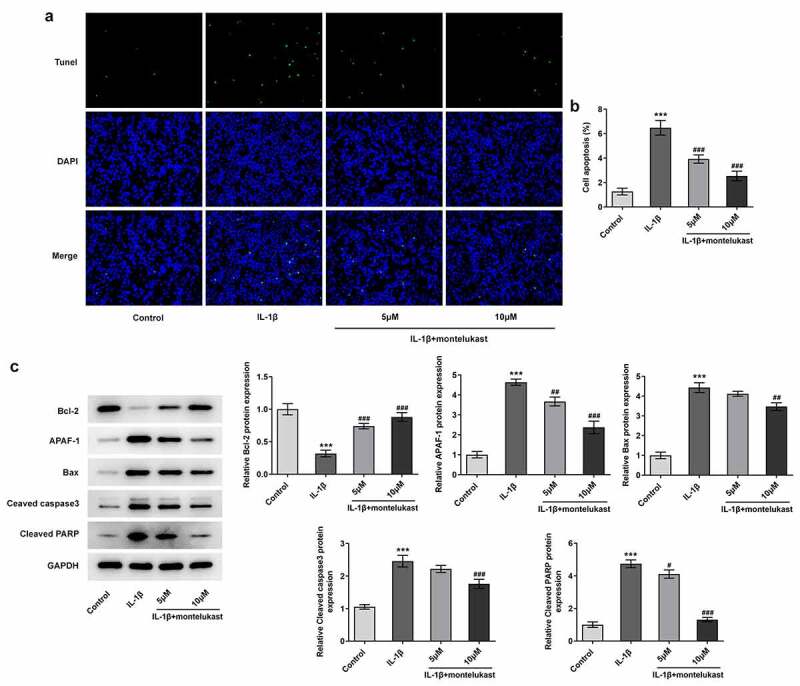
Montelukast attenuates the apoptosis in IL-1β-induced ATDC5 cells. The (a, b) apoptosis and (c) apoptosis-related protein expressions of IL-1β-induced ATDC5 cells were tested by TUNEL, RT-qPCR and western blot under montelukast treatment. ***p < 0.001 Vs control. ^#^p < 0.05, ^##^p < 0.01, ^###^p < 0.001 Vs IL-1β

We next wondered to know whether the effects of montelukast were involved in on oxidative stress and apoptosis in ATDC5 cells in response to IL-1β The cell viability of IL-1β-induced ATDC5 cells treated with montelukast was then assessed by CCK-8. As shown in Figure 2(a), IL-1β decreased the cell viability of ATDC5 cells, which was reversed by montelukast treatment. Results from the detection of ROS, SOD and MDA kits showed decreased ROS and SOD levels and increased MDA level when montelukast was used to treat IL-1β-induced ATDC5 cells (Figure 2(b–c)). Additionally, the protein levels of Nrf2 and HO-1 involved in antioxidation of body were significantly increased in ATDC5 cells with IL-1β stimulation compared with control group. Figure 2(d), while the levels of antioxidant proteins in IL-1β-induced ATDC5 cells was significantly decreased by montelukast treatment. Furthermore, TUNEL and western blot detection reflected that the apoptosis in IL-1β-induced ATDC5 cells was alleviated after montelukast treatment (Figure 3(a–c)). These results suggested that montelukast reversed the oxidative stress and apoptosis in IL-1β-induced ATDC5 cells.

Montelukast improved the expression of KLF2 in IL-1β-induced ATDC5 cells, which restored cell viability damaged by IL-1β

**Figure 4.**
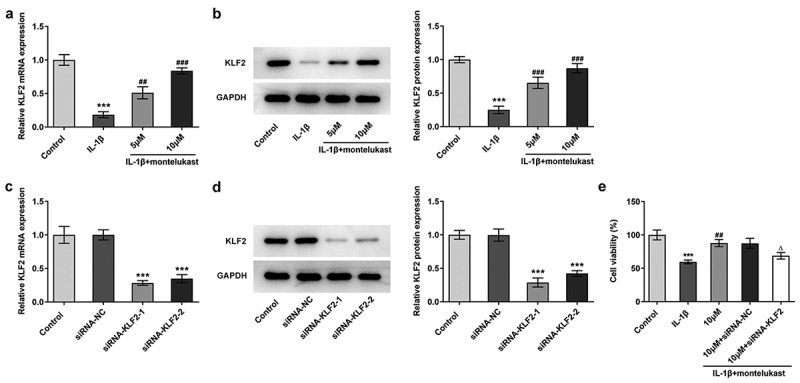
Montelukast promotes the expression of KLF2 in IL-1β-induced ATDC5 cells, which can restore the cell viability damaged by IL-1β. (a–b) The expression of KLF2 in IL-1β-induced ATDC5 cells treated with montelukast was detected by RT-qPCR and western blot. ***p < 0.001 Vs control. ^##^p < 0.05, ^###^p < 0.001 Vs IL-1β. (c) The level of KLF2 in IL-1β-induced ATDC5 cells treated with montelukast after transfection with siRNA-KLF2 was measured by RT-qPCR and western blot. (d) The cell viability in IL-1β-induced ATDC5 cells co-treated with montelukast and siRNA-KLF2 was examined by CCK-8. ***p < 0.001 Vs control. ^##^p < 0.01 Vs IL-1β. ^Δ^P<0.05 Vs 10 µM+siRNA-NC

Montelukast was previously reported to activate KLF2 expression in the treatment of atherosclerosis [28,29]. We further determined how montelukast improved oxidative stress and apoptosis in ATDC5 cells when subjected to IL-1β stimulation. Our previous experiments have demonstrated that KLF2 expression was down-regulated in chondrocytes and OA cartilage tissue, and alleviated the oxidative stress in OA by enhancing KLF2 expression. Thus, we next investigated whether montelukast alleviated apoptosis and oxidative stress via KLF2. Consistent with previous findings, IL-1β inhibited the expression of KLF2, which was reversed by montelukast administration (Figure 4(a,b)). After transfecting with siRNA-KLF2, siRNA-KLF2-1 was used for the following assays due to its better transfection efficiency (Figure 4(c,d)). It was clearly indicated in Figure 4(c) that silencing KLF2 inhibited the reversal effect of montelukast on cell viability of IL-1β-induced ATDC5 cells (Figure 4(e)). Overall, montelukast facilitated the expression of KLF2 in IL-1β-induced ATDC5 cells, which can restore the cell viability damaged by IL-1β.

Montelukast attenuates oxidative stress and apoptosis in IL-1β-induced ATDC5 cells by activating KLF2

**Figure 5.**
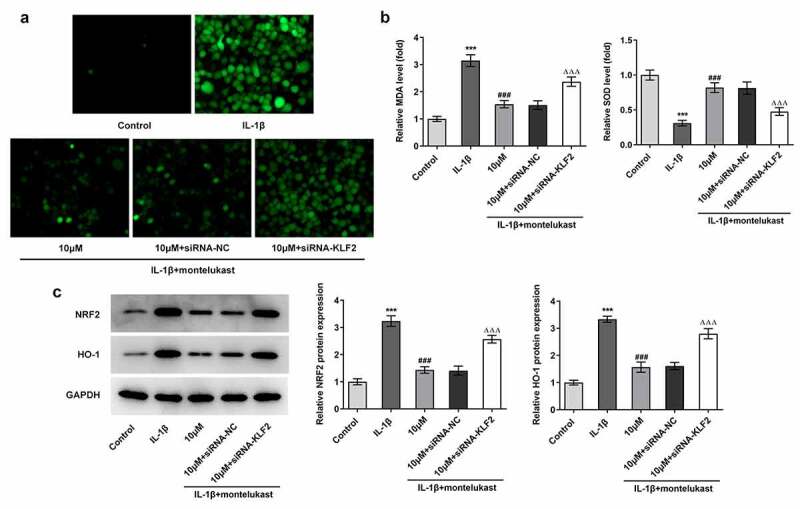
Montelukast attenuates oxidative stress in IL-1β-induced ATDC5 cells by activating KLF2. The (a) ROS, (b) SOD and (c) MDA levels in IL-1β-induced ATDC5 cells co-treated with montelukast and siRNA-KLF2 were measured by corresponding kits. (d) The expressions of antioxidant proteins were measured by western blot in IL-1β-induced ATDC5 cells co-treated with montelukast and siRNA KLF2. ***p < 0.001 Vs control. ^###^p < 0.01 Vs IL-1β. ^ΔΔΔ^P<0.001 Vs 10 µM+siRNA-NC

**Figure 6.**
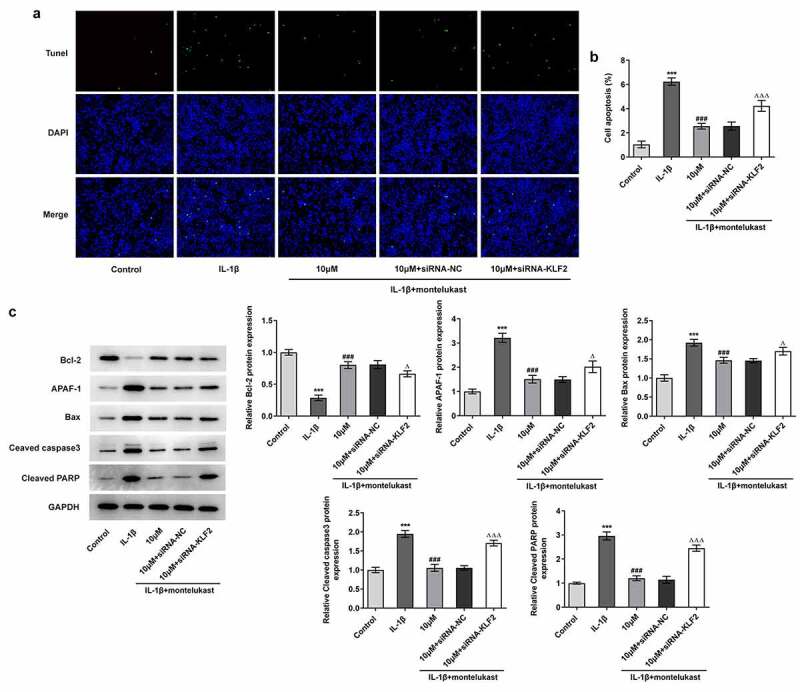
Montelukast attenuates apoptosis in IL-1β-induced ATDC5 cells by activating KLF2. The (a, b) apoptosis level and apoptosis-related protein expressions in IL-1β-induced ATDC5 cells co-treated with montelukast and siRNA-KLF2 were detected by TUNEL, western blot and RT-qPCR. ***p < 0.001 Vs control. ^###^p < 0.01 Vs IL-1β. ^Δ^P<0.05, ^ΔΔΔ^P<0.001 Vs 10 µM+siRNA-NC

To identify the potential mechanism underlying the effect of montelukast in IL-1β-stimulated chondrocytes, siRNA-KLF2 was transfected into IL-1β-induced ATDC5 cells pretreated with montelukast. Notably, IL-1β-induced ATDC5 cells treated with montelukast showed lower levels of ROS and MDA and higher level of SOD together with decreased expression of antioxidant proteins, whereas treatment with siRNA-KLF2 showed marked reversal effects (Figure 5(a–c)). Interestingly, interference of KLF2 abated the restorative effect of montelukast on the apoptosis of IL-1β-induced ATDC5 cells. The apoptosis rate and apoptosis-related proteins levels showed consistent changes after transfection (Figure 6(a–b)). These data suggested that montelukast attenuated oxidative stress and apoptosis in IL-1β-induced ATDC5 cells by activating KLF2.

## Discussion

IL-1β has been used as a critical stimulus for the induction of inflammation and oxidative stress in various chronic inflammatory diseases. Extensive studies have indicated the role of IL-1β in inducing the inflammatory response in OA [30]. In the present study, ATDC5 cells were stimulated by IL-1β to mimic an *in vitro* OA model. Then, different concentration of montelukast was added for the observation of its effects on the cell model. Our results showed that IL-1β induced a high expression of CysLTR1, damaged cell viability, enhanced oxidative stress and alleviated apoptosis of ATDC5 cells. The apoptosis of chondrocytes was also deemed as a key factor in the pathogenesis of OA [31].

KLF members are located in multicellular organisms, where they can regulate a large number of physiological processes [32]. KLF2, an essential factor of this family, participates in a wide range of processes, including cell differentiation, formation of a barrier between the blood and tissues, and regulation of vascular systems [33]. Emerging evidence suggests that the suppression of NF-κB signaling by mediating KLF2 expression, a pathway known to be associated with inflammatory responses, reduces the production of inflammatory cytokines like IL-1β and TNFα, thereby regulating the inflammatory processes in various diseases [32]. Mechanistically, KLF2 relieves bleomycin-induced pulmonary fibrosis and inflammation by the mediation of Activator protein-1 [34]. In this work, IL-1β abated the expression of KLF2, whereas montelukast treatment reversed the effect of IL-1β on ATDC5 cells in a concentration-dependent way. To investigate the involvement of montelukast in KLF2 expression, we constructed the siRNA targeting KLF2 and transfected it into ATDC5 cells induced by IL-1β. The results indicated that siRNA-KLF2 damaged the restorative effects of montelukast on the oxidative stress and promoted the apoptosis of IL-1β-induced ATDC5 cells treated with montelukast. We could see that KLF2 mediates the effects of montelukast against oxidative stress and apoptosis on IL-1β-induced ATDC5 cells. It was reported that extracellular signal-regulated kinase 5 mediated the expression of KLF2 to participate in the anti-inflammatory effects of montelukast [28]. In summary, the present work firstly reveals the potential beneficial role of montelukast relating to the modification of oxidative stress and apoptosis in OA.

## Conclusion

In summary, this work provided important insights that montelukast attenuated oxidative stress and apoptosis in IL-1β-induced chondrocytes by inhibiting CysLTR1 and activating KLF2. The findings of this study also implied that montelukast may be a potential therapeutic strategy against OA development in the future. This study lacks in vivo study to further confirm the effects of montelukast on OA, which is the limit of this study.

## Data Availability

The datasets used and/or analyzed during the current study are available from the corresponding author on reasonable request.

## Highlights

1. Montelukast reduces CysLTR1 and promotes KLF2 expression in response to IL-1β.
2. Montelukast decreases oxidative stress and apoptosis in IL-1β-induced chondrocytes.
3. Montelukast could promote KLF2 expression to resist oxidative stress induced by IL-1β.
4. Montelukast protects chondrocytes from IL-1β-induced apoptosis.
